# Spatiotemporal trajectory of life expectancy and its disparity in China 2000–2030: modelling and prediction

**DOI:** 10.1186/s12889-025-25201-x

**Published:** 2025-11-12

**Authors:** Yuqing Feng, Jinfeng Wang, Naliang Guo, Yue Cai, Qian Yin, Shiyong Wu

**Affiliations:** 1 State Key Laboratory of Resources and Environmental Information System, Institute of Geographic Sciences and Natural Resources Research, Chinese Academy of Sciences, Beijing, China; 2 College of Resources and Environment, University of Chinese Academy of Sciences, Beijing, China; 3 Key Laboratory of Land Surface Pattern and Simulation, Institute of Geographic Sciences and Natural Resources Research, Chinese Academy of Sciences, Beijing, China; 4 Center for Health Statistics and Information, National Health Commission, Beijing, China

**Keywords:** Life expectancy, Tree like evolution trajectory, Physical and socioeconomic determinants, Projection

## Abstract

**Background:**

Life expectancy (LE) is one of crucial metrics of human evolution. However, the evolutionary trajectories of LE in different regions of China and the regional inequalities expected in 2030 are still unclear yet.

**Methods:**

This study collected provincial LE data and relevant explanatory variables for the years of 2000, 2010, 2020 in China. The Geotree method was employed to reconstruct the evolution trajectories of LE, while a multilevel model was used to predict LEs at the provincial levels in the country for the year 2030.

**Results:**

The LE in China exhibited significant geographical pattern, decreasing from the east to the west of the country. LE increases with socio-economic development but is constrained by the natural environment. The physical limitation to LE is significant in western China but are being alleviated with the development of socio-economic conditions. LE will increase in all provinces by 2030, with the overall LE in China reaching 80.05 years (95% confidence interval: 78.93 ~ 81.28), and regional inequalities will diminish.

**Conclusions:**

LE is increasing with the improvement of socioeconomic condition over time; the constraints imposed by the natural environment on LE are being overridden with the improvement of socio-economic conditions.

**Supplementary Information:**

The online version contains supplementary material available at 10.1186/s12889-025-25201-x.

## Introduction

 Life expectancy (LE) serves as a pivotal indicator reflecting population health status and quality of life in a country or region [1, 2]. Its spatiotemporal variation reflects the integrated impacts of various factors such as socio-economic progress, environmental resilience, and public health policies [3–5]. In China, LE has been explicitly adopted as a reference in national health policy formulation. The “Healthy China 2030” Plan, released in 2016 by the State Council, identified LE as a significant metric and delineate specific developmental targets [6, 7].

The spatiotemporal distribution of LE results from the complex interplay of multiple influencing factors. Numerous studies have indicated that improvements in socio-economic factors, such as the economy, education, and healthcare, play a vital role in shaping LE [8–10]. Environmental factors are also recognized as substantial contributors to variations in LE [11, 12]. Evidence suggests that, even after adjusting for socio-economic and healthcare variables, areas endowed with extensive green spaces and temperate climates are correlated with extended LE [13, 14]. Air pollution, one of the major environmental factors affecting public health, results in the deaths of one million people in China each year [9, 15, 16]. In terms of population characteristics, LE disparities between genders, influenced by an amalgamation of biological, social, and environmental factors, signify that females live longer than males [17]. This phenomenon suggests an association between the gender composition of a population and its overall LE. The gross dependency ratio affects LE by influencing the proportion of working-age population and per capita disposable income in a region [18, 19]. Although prior studies have extensively explored the factors influencing LE, the underlying interactive mechanisms among these factors remain inadequately understood.

China has witnessed one of the world’s most rapid increases in LE, from 41.0 years in 1949 to 78 years in 2020, an almost a twofold increase [20]. However, due to significant environmental diversity and uneven socio-economic development across provinces, pronounced interprovincial disparities persist [21]. In 2020, provincial LE varied significantly, ranging from 72.19 years in Tibet to 82.55 years in Shanghai [22]. In the future, how to enhance regional health equity while maintaining improvements in LE remains a major challenge. Forecasting LE at the provincial level for future periods is vital for helping government authorities and decision-makers foresee health risks and resource demands and for formulating pertinent strategies and action plans with greater accuracy [23].

The significant heterogeneity of LE within China offers an ideal context to explore how environmental and socioeconomic factors jointly shape the spatiotemporal evolution of LE. Provinces situated in different natural environments and at varying developmental stages may exhibit distinct trends in LE, while provinces of the same type may display similar developmental patterns. This study investigates the evolutionary trajectories of LE from the perspective of stratified heterogeneity in natural environments and socioeconomic development. The Geotree model, a multidimensional visualization approach grounded in the concept of stratified heterogeneity, represents potential mechanisms and evolutionary processes embedded in high-dimensional data intuitively [24–27].

Therefore, we employed Geotree model to explore the underlying mechanisms underlying the relationship between LE and both environmental and socio-economic factors. Based on this, we constructed a multilevel model to project the LE of 31 provincial-level regions in China by 2030 and to assess regional inequalities. The study aims to provide scientific evidence and policy recommendations for formulating locally tailored strategies to promote health equity.

## Data and materials

### Sociodemographic and environmental determinants of LE

Based on previous literature and data availability, we collected the explanatory variables for LE from four dimensions: socioeconomic development, population characteristics, healthcare resources and environmental exposures. Socioeconomic development across regions was measured in this study by per capita GDP, average educational attainment, and urbanization rate. Healthcare services were measured by the number of physicians per 1,000 people and the proportion of out-of-pocket health expenditure (OOP). Sex ratio and gross dependency ratio were used as indicators of population characteristics. Environmental variables considered in this study included population-weighted elevation, temperature, precipitation, normalized difference vegetation index (NDVI), and PM2.5 concentration. To control temporal confounding, we included “year” as a candidate variable. (Table 1).

**Table 1:** Selection basis of factors affecting life expectancy

Type	Variable	Reference
Socioeconomic development	GDP per capita	[8–10, 14]
	Urbanization rate	
	Average years of schooling	
Healthcare resources	Number of practicing (assistant) physicians per 1,000 population	[28, 29]
	Proportion of out-of-pocket (OOP) health expenditure	
Population characteristics	Gross dependency ratio	[17, 28]
	Sex ratio	
Environmental exposures	Population-weighted elevation	[8, 12–16]
	Population-weighted NDVl	
	Population-weighted PM2.5	
	Population-weighted temperature	
	Population-weighted precipitation	

### LE and socioeconomic data

The provincial LE data for the years 2000, 2010 and 2020 in China were obtained from the China Health Statistical Yearbook. Indicators including urbanization rate, per capita GDP, average years of schooling, OOP, gross dependency ratio, and sex ratio were obtained from national or provincial statistical yearbooks. Elevation data with a spatial resolution of 1 km was obtained from the Resource and Environment Science and Data Center [30]. Population density data with a spatial resolution of 1 km was sourced from WorldPop [31, 32]. The calculation formula for population-weighted elevation ($\:{wDEM}_{p}$) is as follows:

$$\:{wDEM}_{p}={\sum\:}_{i}^{n}{w}_{i,p}\times\:{DEM}_{i,p}$$

$$\:{w}_{i,p}=\frac{{pop}_{i,p}}{{\sum\:}_{i}^{n}{pop}_{i,p}}$$

Herein, *i* represents a raster pixel, *p* denotes a province, *n* is the total number of pixels within the province *p*, $\:{pop}_{i,p}$ denotes the population in pixel *i* of province *p*. $\:{w}_{i,p}$ represents the weight of pixel *i* in province *p*, $\:{DEM}_{i,p}\:$indicates the elevation of pixel *i* in province *p*.

### Future data

Future projections of provincial population by age and sex, education attainment, and urbanization rates were obtained from a widely used SSP spatial population scenario database in China [33]. Future socio-economic data was obtained from Science Data Bank [34]. We collected data from the middle road (SSP2) scenario, which indicates the world will maintain the development of recent decades. The 2030 OOP data are from the “Healthy China 2030” Plan.

## Methods

**Fig. 1:**
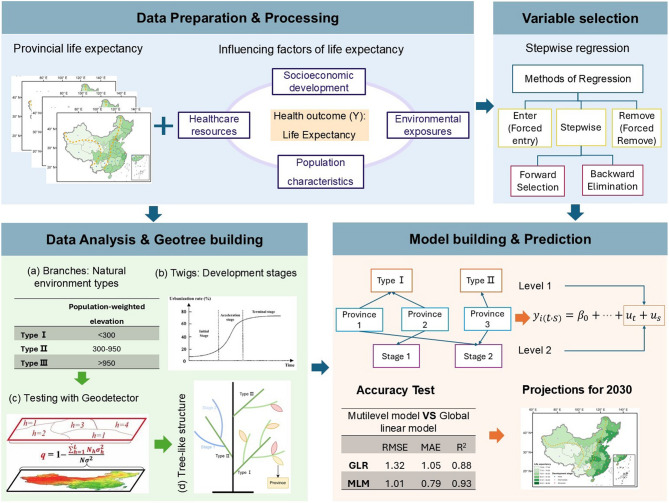
Flow diagram of the analysis based on the Geotree framework, which comprises three components: branches, twigs, and leaves

Based on classifications of provinces according to environmental characteristics and development stages, we employed the Geotree method to reconstruct the LE evolution trajectories of 31 provincial-level administrative divisions. A multilevel model was then developed to estimate LE in 2030 (Fig. 1).

### Geodetector

The stratified heterogeneity of LE was measured using Geodetector’s q-statistic value. The GeoDetector is a linearity free model to quantify the association between the spatial distribution of LE and its influencing factors [35–37]. The q-statistic is defined as:

$$\:q=(1-\:\frac{{\sum\:}_{h=1}^{L}{N}_{h}{\sigma\:}_{h}^{2}}{N{\sigma\:}^{2}})\times\:100\%$$

where $\:h\:(h=1,\:2,\:\dots\:,\:L)$ is the spatial stratification of the influencing factors, $\:{N}_{h}$ and $\:N$ are the numbers of units in the $\:{h}^{th}$ stratum and the whole area, respectively. $\:{\sigma\:}_{h}^{2}$ and $\:{\sigma\:}^{2}$ are variances in LEs in the $\:{h}^{th}$ stratum and the whole area, respectively. The $\:q$ value varies between 0% and 100%, which can be interpreted as deterministic power of the explanatory variable, i.e., the percent of variance of LE explained by an explanatory variable.

### Geotree method

The Geotree model, inspired by the principles of biological evolution, reconstructs the evolutionary trajectory of an observed phenomenon using cross-sectional data. It is particularly applicable to stratified evolutionary processes [24–27]. The structure of the Geotree consists of branches, twigs and leaves, where the branches and twigs represent natural environment types and socioeconomic developmental stages of the leaves (i.e., provinces), respectively. In this study, population-weighted elevation was used to define the branches of the Geotree, reflecting the natural environment of each region. Provinces were grouped into three categories using the natural breaks method. Twigs indicate the development stage of each province, classified according to urbanization rates. According to the Northam curve [38], these stages were defined as initial, intermediate, and advanced. Each province was represented by a leaf on the tree, positioned according to its environmental type and development stage. Detailed classifications are provided in Table 2.

**Table 2:** Indicators of types and development stages of provinces

	Natural environment types	Development stages
	Population-weighted elevation (m)	Urbanization rate (%)
Ⅰ (1)	< 300	< 30
Ⅱ (2)	300–950	30–70
Ⅲ (3)	> 950	> 70

### Multilevel method

Multilevel model (MLM) is suitable for modeling data with a hierarchical structure [39, 40]. The Geotree offers a hierarchical framework for MLM, and in turn, MLM captures the structure embedded within the Geotree. Based on the previously constructed Geotree, we employed a MLM with cross random effects. The model is expressed as follows:

$$\:{y}_{i(t,s)}={\beta\:}_{0}+{\beta\:}_{1}{x}_{i(t,s)}+{u}_{t}+{u}_{s}{+e}_{i(t,s)}$$

$$\:{u}_{t}\:\sim\:N\left(0,{\sigma\:}_{u\left(t\right)}^{2}\right),\:{u}_{s}\:\sim\:N\left(0,{\sigma\:}_{u\left(s\right)}^{2}\right),{e}_{i(t,s)}\:\sim\:N\left(0,{\sigma\:}_{e}^{2}\right)$$

Here, *t* and *s* represent the environmental type and developmental stage of province *i*, respectively, and $\:{y}_{i(t,s)}$ represents its LE. $\:{\beta\:}_{0}$ is intercept, $\:{x}_{i(t,s)}$ indicates the influencing factors of LE and $\:{\beta\:}_{1}$ is the corresponding coefficient. $\:{u}_{s}\:$and $\:{u}_{t}\:$represent the random effects of environmental type *t* and development stage *s*, respectively. $\:{e}_{i(t,s)}$ is the residual error term. We employed a stepwise regression approach to select variables for inclusion in the model. Ultimately, five explanatory variables were retained in the MLM: per capita GDP, average years of schooling, OOP, gross dependency ratio, and sex ratio. This model was employed to forecast future LE across China’s provinces.

## Results

### Natural environment types and socioeconomic development stages

**Fig. 2:**
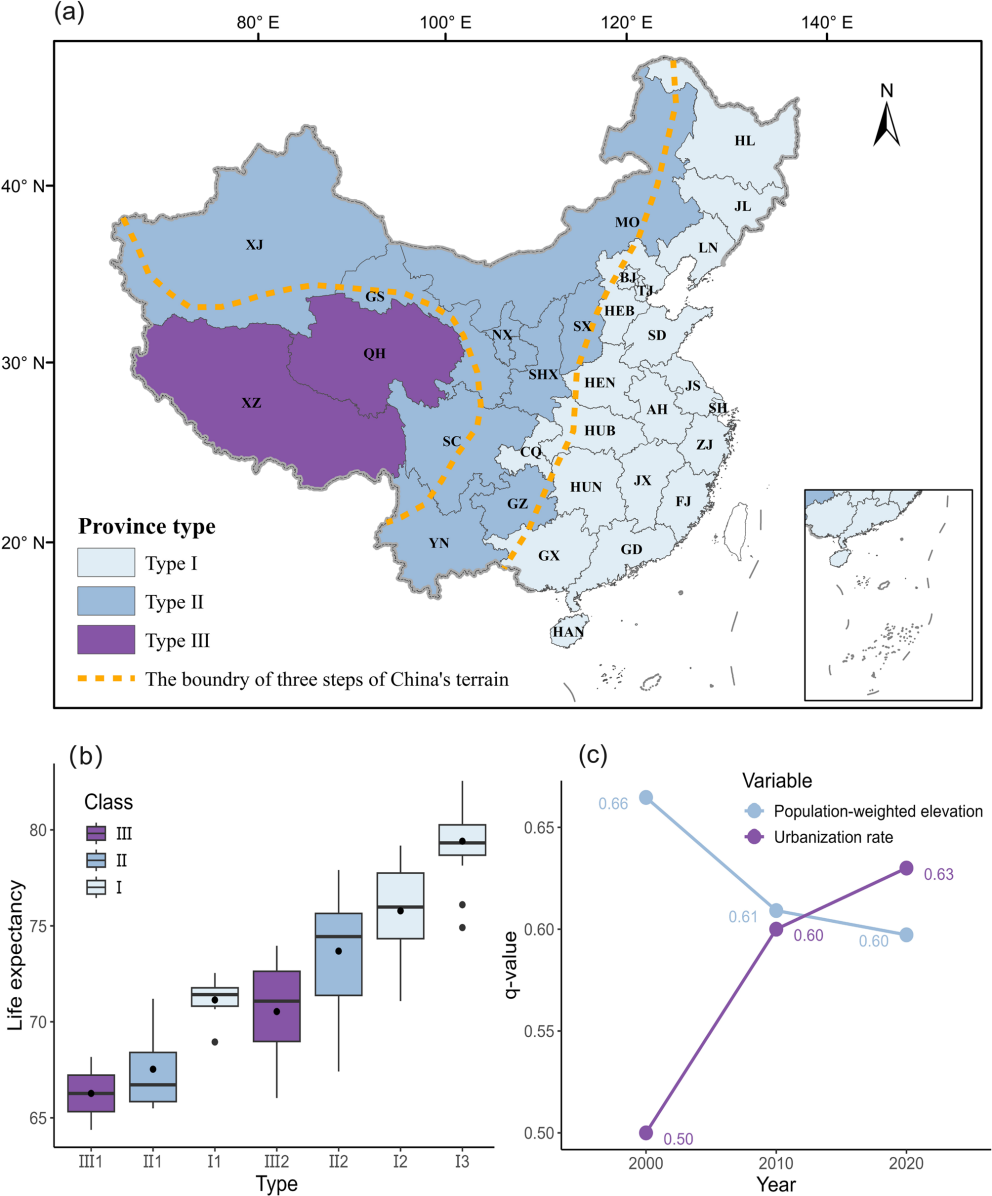
**a** Geographical distribution of the types of provinces; **b** LE in different natural environment types and development stages, in which natural environment types are denoted by Ⅰ, Ⅱ, Ⅲ, and development stages are denoted by 1, 2, 3, respectively; **c** q-values of LE explained by population-weighted elevation and urbanization rate

The distribution of natural environment type is shown in Fig. 2a. Provinces classified as Type I are primarily located in the eastern China, featuring plains with elevations below 500 m. Around 75.8% of the national population is concentrated in this region. Provinces classified as Type II are mainly located in the central and northwest regions of China, with elevations ranging from 1000 to 2000 m. Approximately 23.6% of the population lives in this area. Provinces classified as Type III include Tibet and Qinghai, situated in the Qinghai-Tibet Plateau, with an average elevation exceeding 4000 m. These regions have a sparse population, with only 0.6% of people residing there.

Significant variations in LE are observed across different natural environment types and developmental stages (Fig. 2b). Within the same natural environment type, provinces at higher developmental stages tend to exhibit higher LE. Similarly, at the same developmental stage, provinces with a Type I natural environment have the highest LE, followed by Types II and III.

Both natural environment type and development stage exhibit strong explanatory power for LE (Fig. 2c). From 2000 to 2020, the q-value of natural factors declined, while the q-value of urbanization rose. This suggests that over time, the impact of the natural environment on LE has diminished, and the influence of social development has grown, indicating an improvement in human adaptive capabilities to the environment.

### Spatial distribution and tree structure of life expectancy

**Fig. 3:**
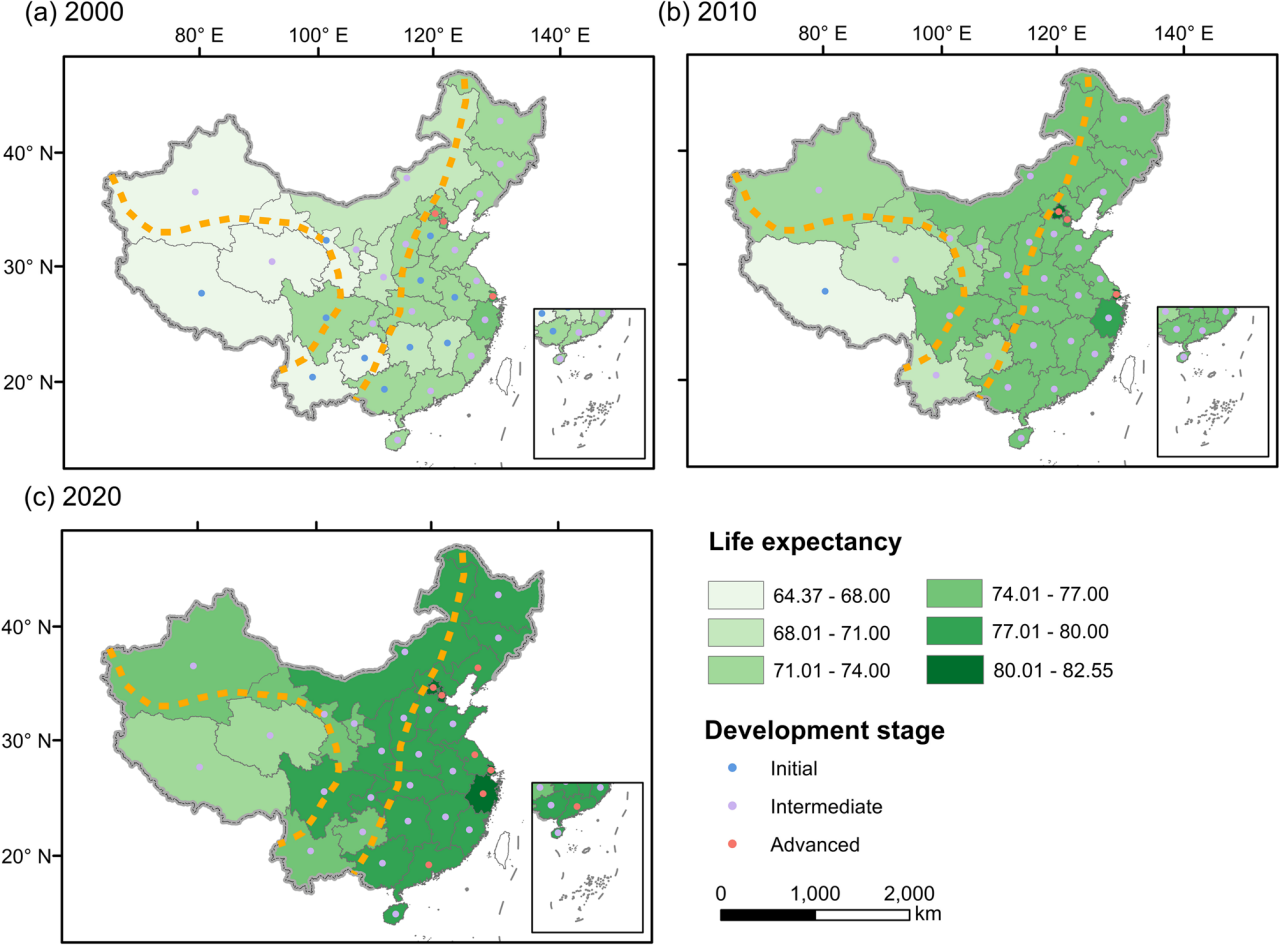
Spatial distribution of LE and development stages by province in **a** 2000, **b** 2010 and **c** 2020

LE has increased steadily over time. By 2020, all provinces had reached the intermediate or advanced stage, and provinces at advanced stages exist only in regions with type I natural environment. Spatially, LE demonstrates a gradual decrease from east to west, and over time, this pattern continues to persist (Fig. 3).

**Fig. 4:**
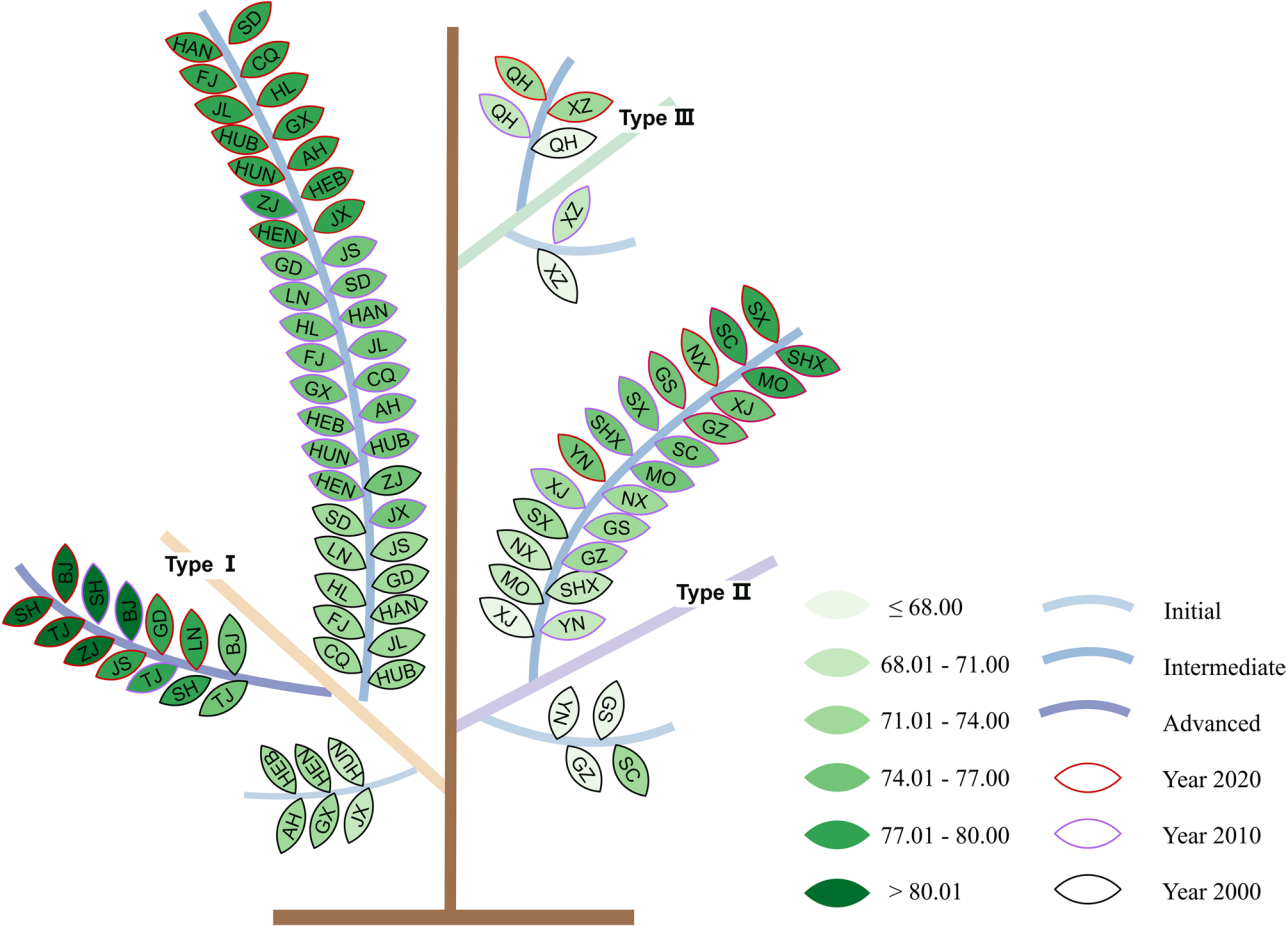
Geotree of LE in 2000, 2010, 2020. (BJ: Beijing, TJ: Tianjin, HEB: Hebei, SX: Shanxi, Inner Mongolia: MO, LN: Liaoning, JL: Jilin, HL: Heilongjiang, SH: Shanghai, JS: Jiangsu, ZJ: Zhejiang, AH: Anhui, FJ: Fujian, JX: Jiangxi, SD: Shandong, HEN: Henan, HUB: Hubei, HUN: Hunan, GD: Guangdong, GX: Guangxi, HAN: Hainan, CQ: Chongqing, SC: Sichuan, GZ: Guizhou, YN: Yunnan, XZ: Tibet, SHX: Shaanxi, GS: Gansu, QH: Qinghai, NX: Ningxia, XJ: Xinjiang)

The natural environment type, developmental stage, and LE exhibit a similar spatial pattern, indicating significant stratified heterogeneity and coupling relationships. We further mapped the three variables into a hierarchical tree-like structure and built a Geotree, which provides an “attribute-space coordinate system”. Leaf color variations within branches indicate differences in LE across natural environment types (Fig. 4). On twigs, as the socio-economic development, leaf colors shift from yellow to green, reflecting a gradual rise in LE. These trends are substantiated by the boxplots in Fig. 2.

### Prediction of LE in 2030

**Fig. 5:**
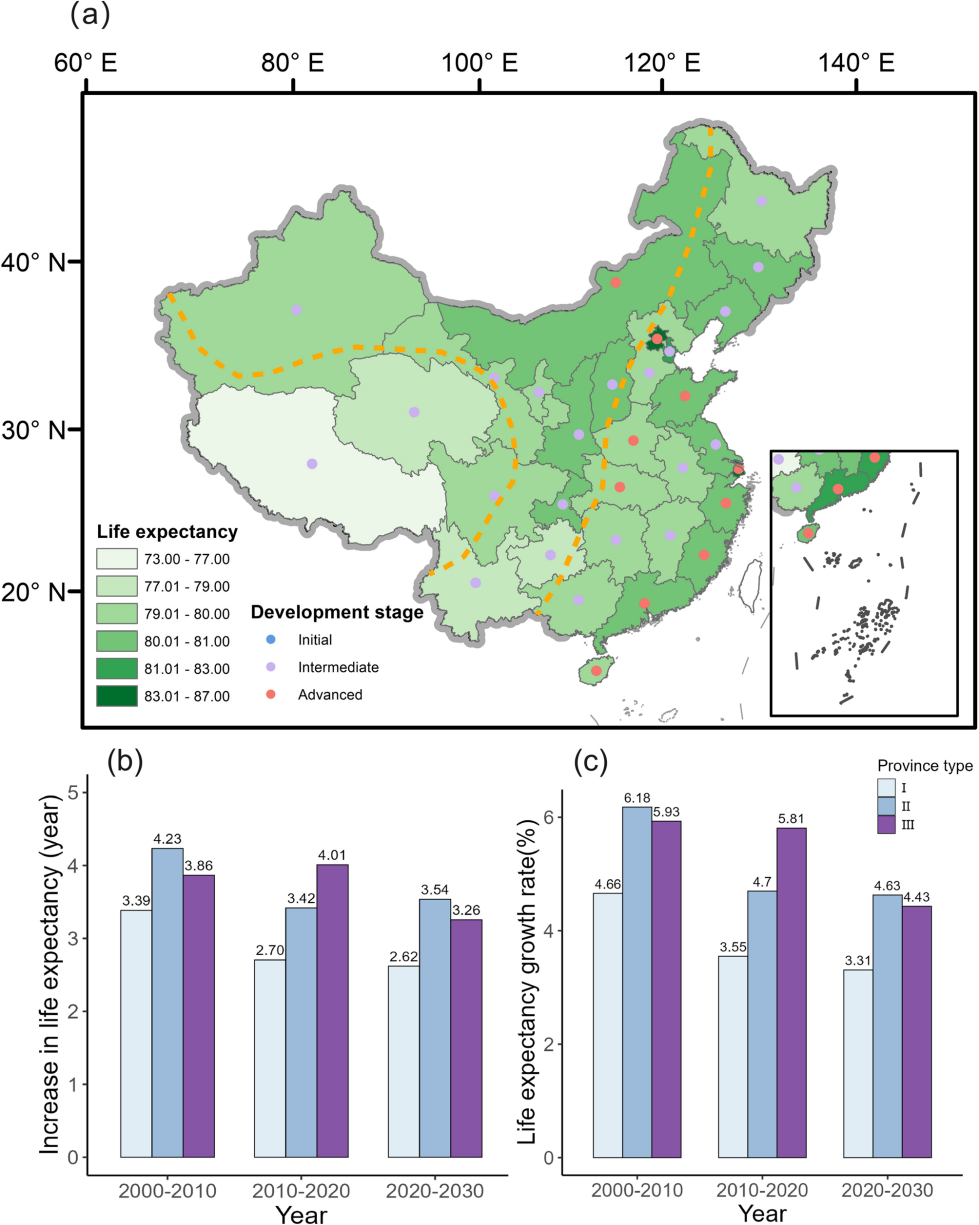
**a** Spatial distribution of LE and development stages by province in 2030; **b** Increase in life expectancy per decade, 2000–2030; **c** Life expectancy growth rate per decade, 2000–2030

Based on the structure of the Geotree, we developed a multilevel model and compared it with generalized linear regression. Cross-validation results (Table S2) show that multilevel model more effectively captures the evolution of LE under different types of natural environment and developmental stages, and it was used to estimate LE for each province in 2030. LE in China will reach 80.05 (95% confidence interval: 78.82 ~ 81.28) years by 2030, an increase of 2.12 years. In the eastern regions, the provincial LE remains at the forefront. The spatial pattern of higher in the east and lower in the west persists and no province remains in the first stage of development (Fig. 5a).

From 2000 to 2030, life expectancy (LE) increased in all provinces, although the growth rate showed a declining trend over time. Type I provinces exhibited the lowest average growth rate, with an average increase of 2.62% (3.31 years) from 2020 to 2030 (Fig. 5b). Next were the provinces in type III, with an increase of 3.26 years (4.43%), while type II provinces have the highest growth rate (3.54 years, 4.63%). The inequality in LE among regions is gradually decreasing by 2030 (Fig. 5c).

## Discussion

This study constructed the spatiotemporal trajectory of provincial LE in China and forecasted LE across provinces for the year 2030. The findings indicate that LE in China is shaped by the combined influence of natural and socio-economic factors. From 2000 to 2020, China’s LE steadily increased, which is closely associated with improvements in regional socio-economic development and healthcare conditions. Over time, the spatial pattern of LE has become increasingly influenced by socio-economic conditions, with the role of the natural environment gradually weakening. Nevertheless, significant health disparities persist across regions. By 2030, China’s LE is projected to reach 80.05 years, with a narrowing of health disparities among different types of provinces.

The natural environment, including factors such as climate, terrain, and vegetation, is one of the most important influences on the health of the population. Elevation influences mortality and potential lifespan by altering environmental factors such as temperature, atmospheric pressure, oxygen concentration, and ultraviolet radiation intensity [12]. In this study, provinces were classified into three types based on population-weighted elevation, which serves as a proxy for the natural environment. The spatial distribution of these types corresponds closely with the three steps of China’s terrain. By calculating the q-values of population-weighted elevation and visualizing the developmental trajectory of LE using a tree-based structure, we identified natural environment as one of the primary influencing factors explaining spatial heterogeneity in LE [11, 12].

Each type of natural environment exhibits distinct environmental characteristics. Tibet and Qinghai, characterized by natural environment type III, are primarily located in the Qinghai-Tibet Plateau, with elevations exceeding 4000 m. The primary environmental factors affecting health in this region include low levels of oxygen in the air and cold temperatures. A series of studies has demonstrated a close association between hypoxic and the onset of tumors [41, 42], cardiovascular diseases [42], diabetes, and neurodegenerative diseases [43]. Prenatal hypoxia in pregnant women may impair fetal development, leading to underdevelopment of the brain [41], heart [44], nervous system, and other organs [45, 46]. Conversely, environments with higher oxygen concentrations have been associated with longer life expectancy [47]. Over the past 50 years, the annual average temperature on the Qinghai-Tibet Plateau has been 5.85 degrees Celsius [48]. Numerous studies have reported an association between low temperatures and increased risks of various cardiovascular, respiratory, and other diseases [49–52]. The majority of the mortality burden related to non-optimal temperatures is attributed to the contribution of cold [53].

In Type I provinces, population distribution is closely linked to both natural environments and socioeconomic conditions. These provinces have the highest population density among all regions. On one hand, this area lies predominantly southeast of the Hu Huanyong Line, situated in the first step of China’s terrain. The natural environment in these areas is characterized by a monsoon climate with annual rainfall exceeding 1000 mm (Table S2). People generally prefer residing in areas with flat terrain and favorable climates, and approximately 75% of China’s population living in regions characterized by type Ⅰ [54, 55]. On the other hand, these geographic advantages are further converted into developmental potential. Type I provinces exhibit higher urbanization rates and greater density of healthcare resources than the other two types (Table S2). The positive feedback between population and economic development continuously attracts people and resource inputs, which may exacerbate regional imbalances in healthcare resource allocation. With increasing population in Type I provinces, they exhibit higher PM2.5 levels compared to other province types, highlighting the environmental health risks associated with the urbanization process. The remaining areas are classified as type Ⅱ, characterized mainly by arid conditions in the northwest and mountainous terrain in the south. The heterogeneity of the natural environment influences population distribution and, to some extent, shapes patterns of regional development and health resource allocation, underscoring the scientific necessity of classifying provinces based on environmental characteristics.

Socioeconomic development serves as the driving force for the improvement of LE. Significant disparities in LE exist among different income groups, with noticeably higher mortality rates in socioeconomically disadvantaged populations compared to those with higher socioeconomic status [56, 57]. LE demonstrates a significant positive correlation with indicators reflecting socioeconomic development, such as income, healthcare levels, and educational attainment [8–10, 58, 59]. Socioeconomic development signifies the improvement in people’s economic, educational, and healthcare levels, enhancing their adaptability to the natural environment and thereby promoting a reduction in mortality rates.

The dominant factors differ at different stages of the evolution of LE. The urbanization rate can be used as a proxy for the stage of regional development. In the early stages of urbanization, the economy is primarily agrarian, and healthcare and education systems are underdeveloped; at this point, LE is largely determined by the local natural environment. In the intermediate, the urbanization process accelerates, and human adaptability to the environment gradually improves, diminishing the impact of the natural environment on LE. In the advanced, socioeconomic factors become the primary influencers of LE. LE at different stages of development exhibits significant variations (Fig. 2b), with provinces at higher development stages having correspondingly higher LE.

It is crucial to note that human life is consistently exposed to the natural environment. At every stage of development, the impact of the natural environment on LE cannot be eliminated; in other words, the impact of the natural environment on LE persists. LE undergoes categorized development evolution under the influence of natural environment and socio-economic conditions. While the characteristics of the natural environment impose constraints on LE development, improvements in socio-economic conditions can mitigate these limitations. The stratification of LE across natural environment types and developmental stages could facilitate the identification of populations at higher health risk, enables the formulation of targeted policy improvements and resource support.

Over the past three decades, there has been a steady increase in LE in China, and it is projected to reach the Healthy China 2030’ goal of 79.0 years by 2030, which is consistent with findings from previous studies [23]. Since 1990, China has experienced a transition in epidemiological cause-of-death patterns from communicable to non-communicable diseases, and LE at birth has increased significantly in all provinces [60]. By the year 2030, LE in China is projected to increase to 80.05. Yet a spatial pattern persists with a gradual decrease from east to west. The persisting inequality in LE among provinces remains a challenge. Based on the structure of Geotree, the sources of inequality in LE can be broadly categorized into natural (non-intervenable) and social development (intervenable), with social development encompassing healthcare, education, and economic aspects. When social development reaches a certain level, apart from individual behavior, regional differences in LE are primarily due to local environmental characteristics. Therefore, by enhancing healthcare, education, and other levels, regional inequalities can be reduced. To enhance overall health and equity, in addition to advancing socioeconomic development, differentiated strategies should be implemented. Type I provinces should prioritize the prevention and control of environmental health risks associated with urbanization, whereas Type III regions should strengthen the accessibility of primary healthcare services.

This study has several strengths. Firstly, by applying the Geotree method, this study analyzes the spatio-temporal evolution of LE across China, facilitating a deeper understanding of the roles played by various determinants in its evolution. Secondly, our findings recommend strategies for promoting health can be proposed from both natural environment and social development perspectives, contributing to the achievement of equity objectives outlined in “Healthy China 2030”. Provinces of the same type can draw on the experiences of those at a higher developmental stage in the same category, exploring strategies for health improvement that are suitable for their own context. Nevertheless, this study is constrained by data availability, which limited the inclusion of individual-level determinants such as genetics, dietary intake, and physical activity. Additionally, the analysis is based on provincial-level averages, which may mask heterogeneity at finer administrative levels. Finally, it should be noted that the future scenario data used in this study were developed based on pre-COVID-19 conditions. Therefore, the forecasted LE may not reflect potential long-term impacts of the COVID-19 pandemic on mortality or socio-economic patterns in China. Furthermore, the model does not account for unexpected shocks, such as the pandemic, that may affect population health, which is a common limitation. Future research should aim to incorporate a broader set of indicators and more granular data to enhance the construction of China’s LE evolutionary framework.

## Conclusions

This study reconstructs the provincial LE evolution trajectory in China from 2000 to 2020 and predicts it for 2030. The results indicate a spatial pattern of declining LE from east to west and significant stratified heterogeneity. LE evolves with the development stages of society, while being constrained by natural environment factors. By 2030, China’s LE is projected to reach 80.05 years, achieving the ‘Healthy China 2030’ goal, with regional inequalities also gradually decreasing. The western regions of China need to enhance socio-economic development and healthcare infrastructure to overcome the constraints imposed by natural environments.

## Supplementary Information

Supplementary Material 1.

## Data Availability

The datasets supporting the conclusions of this article are included within the article. Additional data related to this paper may be requested from the authors.
