# Supplementary material for: Spatiotemporal trajectory of life expectancy and its disparity in China 2000–2030: modelling and prediction
Source: BMC Public Health. 2025 Nov 12;25:3911. doi: 10.1186/s12889-025-25201-x (PMC12613653; doi:10.1186/s12889-025-25201-x)
Supplement: Supplementary file 1 — Supplementary Material 1. [file 12889_2025_25201_MOESM1_ESM.docx]

# Supplementary file

**Spatiotemporal trajectory of life expectancy and its disparity in China 2000 - 2030：Modelling and prediction**

Yuqing Feng^1,2^; Jinfeng Wang ^1,2^*; Naliang Guo ^1,2^, Yue Cai^3^, Qian Yin^1^, Shiyong Wu^3^*

^1^ State Key Laboratory of Resources and Environmental Information System, Institute of Geographical Sciences and Natural Resources Research, Chinese Academy of Sciences, Beijing, China.

^2^ College of Resources and Environment, University of Chinese Academy of Sciences, Beijing, China.

^3^ Center for Health Statistics and Information, National Health Commission, Beijing, China.

* Corresponding Author

**Corresponding Author: Professor Jinfeng Wang**

State Key Laboratory of Resources and Environmental Information System, Institute of Geographical Sciences and Natural Resources Research, Chinese Academy of Sciences, No. 11, Datun Road, Chaoyang District, Beijing, China

E-mail: wangjf@lreis.ac.cn

**Corresponding Author: Professor Shiyong Wu**

Center for Health Statistics and Information, National Health Commission,

No.1 Xizhimenwai South Road, Xicheng District, Beijing, China

E-mail: wusy99@126.com

Table S1 Descriptive statistics of variables

| Variable | unit | Mean | Standard deviation | Coefficient of variation | Min | Quantile | | | IQR | Max |
| --- | --- | --- | --- | --- | --- | --- | --- | --- | --- | --- |
|  |  |  |  |  |  | p25 | p50 | p75 |  |  |
| Life expectancy | year | 74.68 | 3.86 | 5.17 | 64.37 | 72.23 | 74.92 | 77.79 | 5.56 | 82.55 |
| GDP per capita | yuan | 33496 | 27425 | 82 | 5005 | 12923 | 25308 | 44031 | 31109 | 145640 |
| Urbanization rate | % | 51.38 | 17.28 | 33.64 | 19.30 | 38.50 | 51.90 | 62.53 | 24.03 | 89.30 |
| Average years of schooling | year | 8.35 | 1.59 | 19.09 | 3.00 | 7.26 | 8.20 | 9.73 | 2.47 | 12.64 |
| Sex ratio | % | 105.77 | 2.97 | 2.80 | 99.69 | 103.67 | 105.69 | 107.33 | 3.66 | 114.52 |
| Gross dependency ratio | % | 40.53 | 8.26 | 20.38 | 20.95 | 35.01 | 41.35 | 46.69 | 11.68 | 57.79 |
| Proportion of out-of-pocket (OOP) health expenditure | % | 39.01 | 14.90 | 38.20 | 7.16 | 28.25 | 35.11 | 51.94 | 23.69 | 65.98 |
| Number of practicing (assistant) physicians per 1,000 population | person | 2.27 | 0.86 | 37.80 | 0.97 | 1.56 | 2.13 | 2.77 | 1.21 | 5.24 |
| Population-weighted elevation | meter | 286.99 | 422.30 | 147.15 | 2.83 | 31.57 | 83.50 | 391.99 | 360.42 | 1951.87 |
| Population-weighted temperature | ℃ | 6.95 | 2.80 | 40.31 | 1.38 | 4.84 | 7.53 | 8.66 | 3.81 | 12.57 |
| Population-weighted precipitation | mm | 957.22 | 511.98 | 53.49 | 126.73 | 521.85 | 837.65 | 1337.18 | 815.33 | 2197.26 |
| Population-weighted NDVI | / | 0.30 | 0.10 | 32.46 | 0.15 | 0.21 | 0.30 | 0.37 | 0.16 | 0.47 |
| Population weighted PM2.5 | µg/m³ | 48.72 | 14.22 | 29.19 | 19.73 | 37.96 | 48.46 | 58.48 | 20.52 | 82.69 |

Table S2. Mean values of natural and socio-economic variables for each province type from 2000 to 2020.

| Province types | GDP per capita(yuan) | Average years of schooling(year) | Number of practicing (assistant) physicians per 1,000 population(/1000) | Elevation(mteter) | Temperature(℃) | Precipitation(mm) | PM25(µg/m³) | Urbanization rate(%) |
| --- | --- | --- | --- | --- | --- | --- | --- | --- |
| Ⅰ | 39092.46 | 8.75 | 5.67 | 287.10 | 14.66 | 1183.10 | 42.07 | 56.27 |
| Ⅱ | 24011.66 | 8.00 | 5.23 | 1611.61 | 9.11 | 661.29 | 37.50 | 43.88 |
| Ⅲ | 20210.53 | 6.00 | 4.97 | 4392.82 | -1.16 | 470.34 | 31.86 | 36.21 |

Table S3. Overall accuracy of MLM and GLR model results obtained for trained and tested data.

|  | Trained | | | Tested | | |
| --- | --- | --- | --- | --- | --- | --- |
|  | RMSE | MAE | R^2^ | RMSE | MAE | R^2^ |
| GLR | 1.32 | 1.05 | 0.88 | 1.42 | 1.13 | 0.86 |
| MLM | 1.01 | 0.79 | 0.93 | 1.14 | 0.88 | 0.91 |

Table S4 Model parameters before and after adding the Year variable

| Predictors | Without Year | | | | With Year | | | |
| --- | --- | --- | --- | --- | --- | --- | --- | --- |
|  | Estimates | 95% confidence interval | p value | VIF | Estimates | 95% confidence interval | p value | VIF |
| Intercept | 72.27 | 62.86 – 81.68 | <0.001 |  | 72.07 | 62.76 – 81.38 | <0.001 |  |
| Year |  |  |  |  | 0.08 | -0.01 – 0.17 | 0.067 | 9.12 |
| Average years of schooling | 1.43 | 1.13 – 1.74 | <0.001 | 2.86 | 1.15 | 0.74 – 1.57 | <0.001 | 5.22 |
| OOP | -0.04 | -0.07 – -0.02 | <0.001 | 1.93 | -0.02 | -0.05 – 0.01 | 0.111 | 4.07 |
| GDP per capita | 0.15 | -0.02 – 0.33 | 0.091 | 3.17 | 0.15 | -0.02 – 0.33 | 0.085 | 3.08 |
| Sex ratio | -0.08 | -0.16 – 0.00 | 0.052 | 1.03 | -0.07 | -0.14 – 0.01 | 0.104 | 1.06 |
| Gross dependency ratio | -0.03 | -0.06 – 0.00 | 0.068 | 1.04 | -0.04 | -0.08 – -0.01 | 0.015 | 1.35 |
| AIC | 325.205 |  |  |  | 328.295 |  |  |  |

| (a)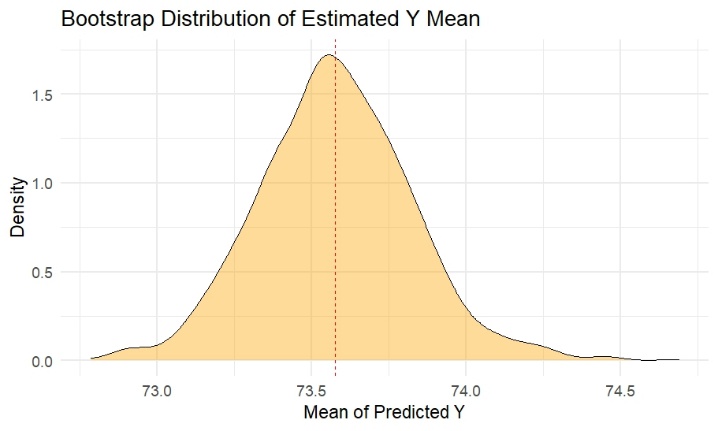 | (b)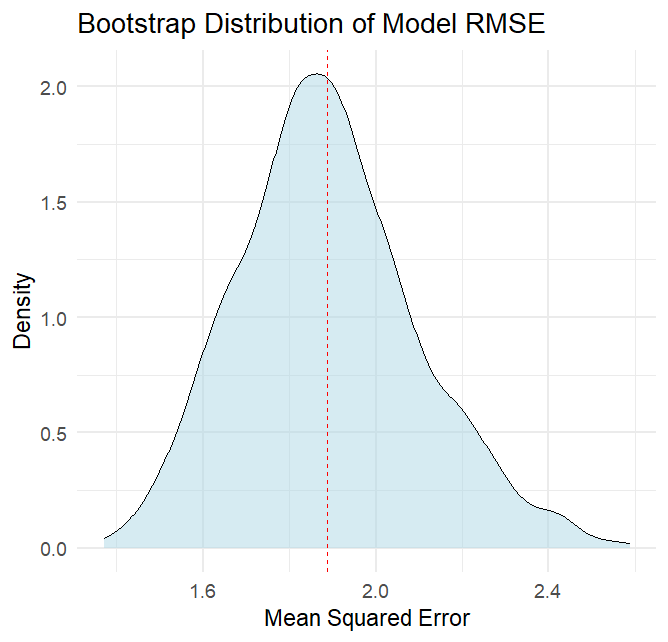 |
| --- | --- |
| (c)  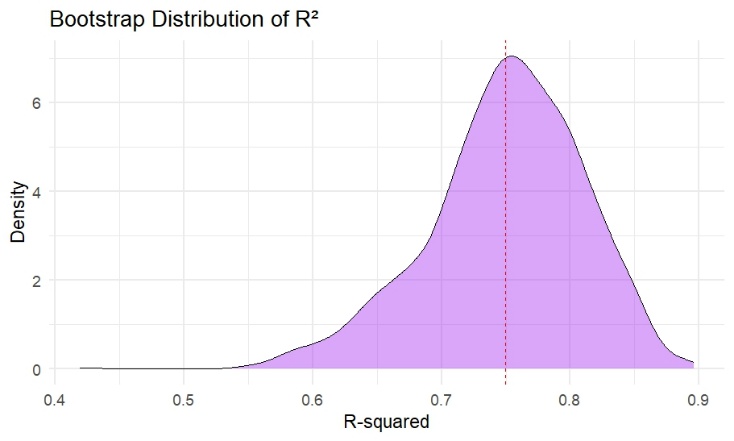 | |

Figure S2 Distributions of Predicted Values, RMSE, and R² from 1,000 Bootstrap Replications
